# Bibliometric analysis of studies on the treatment of hemifacial spasm

**DOI:** 10.3389/fneur.2022.931551

**Published:** 2022-09-01

**Authors:** Li-Jun Fang, Chen-Yao Wang

**Affiliations:** ^1^The Third Clinical College of Zhejiang Chinese Medical University, Hangzhou, China; ^2^Department of Acupuncture, Third Affiliated Hospital of Zhejiang Chinese Medical University, Hangzhou, China

**Keywords:** hemifacial spasm, treatment, bibliometric analysis, VOSviewer, WoS database

## Abstract

**Objective:**

Hemifacial spasm (HFS) is a common neurological disorder of the brain, which is difficult to treat. Most patients are distracted by it and are unable to work or study normally, which seriously affects their physical and mental health. However, there are a few bibliometric studies on it. This paper searched the articles on HFS using a bibliometric approach.

**Method:**

Articles about HFS were retrieved from the Web of Science (WoS) Core Collection database. We employed the Visualization of Similarities (VOS)viewer to analyze these publications.

**Results:**

A total of 645 reviews or articles in English were retrieved from WoS. In this study, we found that the number of publications showed a rising trend and China is the most active in searching the treatment of HFS. About keywords, neurosciences and neurology was searched (422 times) keyword, followed by hemifacial spasm (420 times) and surgery (320 times). By assessing the organizations, Shanghai Jiao Tong University published the most papers (8.68%), followed by Sungkyunkwan University (3.26%) and Baylor College Medicine (2.64%). A total of 247 journals have delivered publications on the treatment of HFS, *World Neurosurgery* (44 papers) published the largest number of articles.

**Conclusion:**

The annual publications have increased with a fluctuating tendency. More researchers were taking an interest in HFS. This study helped us find out the hotspot and trend in research about facial spasm treatment.

## Introduction

Hemifacial spasm (HFS) is a common neurological disorder of the seventh cranial nerve characterized by the involuntary twitching or contractions of the facial muscles (1), seriously, difficulty in opening eyes, mouth deviation, tinnitus, and so on. In most cases, HFS's etiology is the facial nerve compressed by vessels in the pontocerebellar horn region, rare cases are caused by malignant tumors, inflammation, or others (2). About 30% of patients with peripheral facial palsy are left with sequelae, one of which is facial muscle spasm (3). Some scholars (4, 5) hold the opinion that a measure of vascular compression causes demyelination of the nerve root, inducing transmembrane sodium channels on the axon; under a number of trigger factors, such as transient fluctuations in blood pressure or pulse, these voltage-gated channels reach the threshold and eventually generate conductible action potentials from the damaged nerve. In addition, HFS affects ~10 per 100,000 people, mainly women, with a peak age of 40–50 years old (6). This disease makes patients anxious and depressed, affecting the quality of their life.

There are two types of HFS: typical facial spasm and atypical spasm. A typical facial spasm is a spasm that starts in the eyelids and progresses downward to the lower facial muscles, such as the expression muscles of the cheek, whereas an atypical facial spasm is a spasm that starts in the lower facial muscles and progresses upward to the eyelids and frontalis. Atypical facial spasms are uncommon in clinical practice (7).

**Figure 1:**
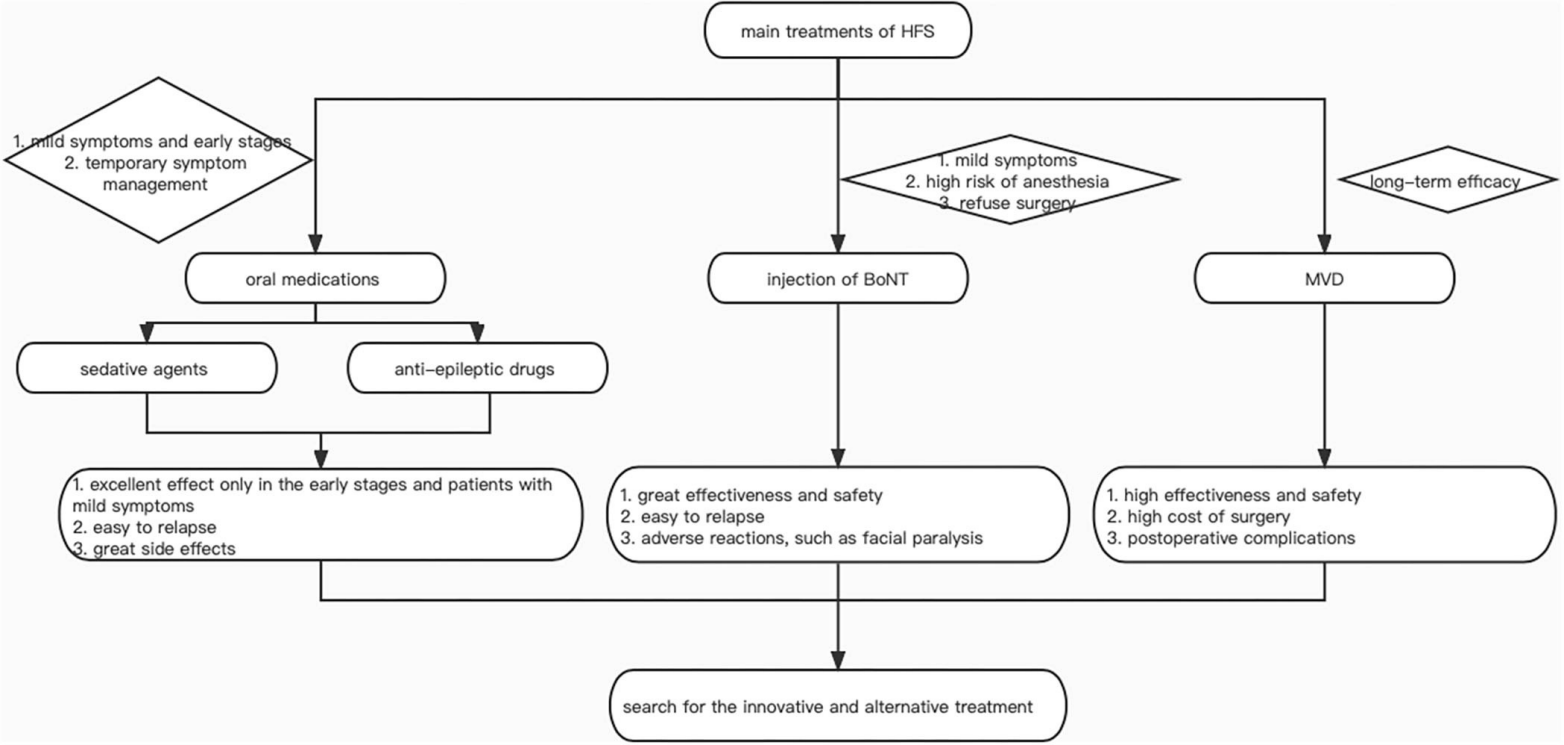
The decision-making flow chart of treatment.

In clinical practice, for the treatment of HFS, there are drug therapy, injections of Botulinum Toxin (BoNT) (8), microvascular decompression (MVD) (9), and so on. It is known that medication is useful for early improvement of symptoms, but long-term curative effects are poor, BoNT is the most commonly used non-operative treatment for HFS, while MVD is a safe and effective treatment for it through removing the offending artery from the facial nerve with a good exposure to relieve spasm (10, 11) (Figure 1). Nevertheless, rare bibliometric analyses focus on the treatment of HFS and there is not a systematic cognition of it.

Bibliometric analysis is a quantitative way to analyze a large number of studies using mathematical and statistical tools in special areas (12). Li et al. (13) did a bibliometric analysis on neuropathy and Goo et al. conducted a research on facial palsy using the bibliometric method (14). Zhang et al. utilized the bibliometric method to know more about acupuncture for analgesia (15). Wang et al. wrote an article about Middle East Respiratory Syndrome using Visualization of Similarities (VOS)viewer (16).

This study aimed to employ a bibliometric analysis on the treatment of HFS *via* VOSviewer to learn about the trends and hotspots.

## Materials and methods

### Data collection

**Figure 2:**
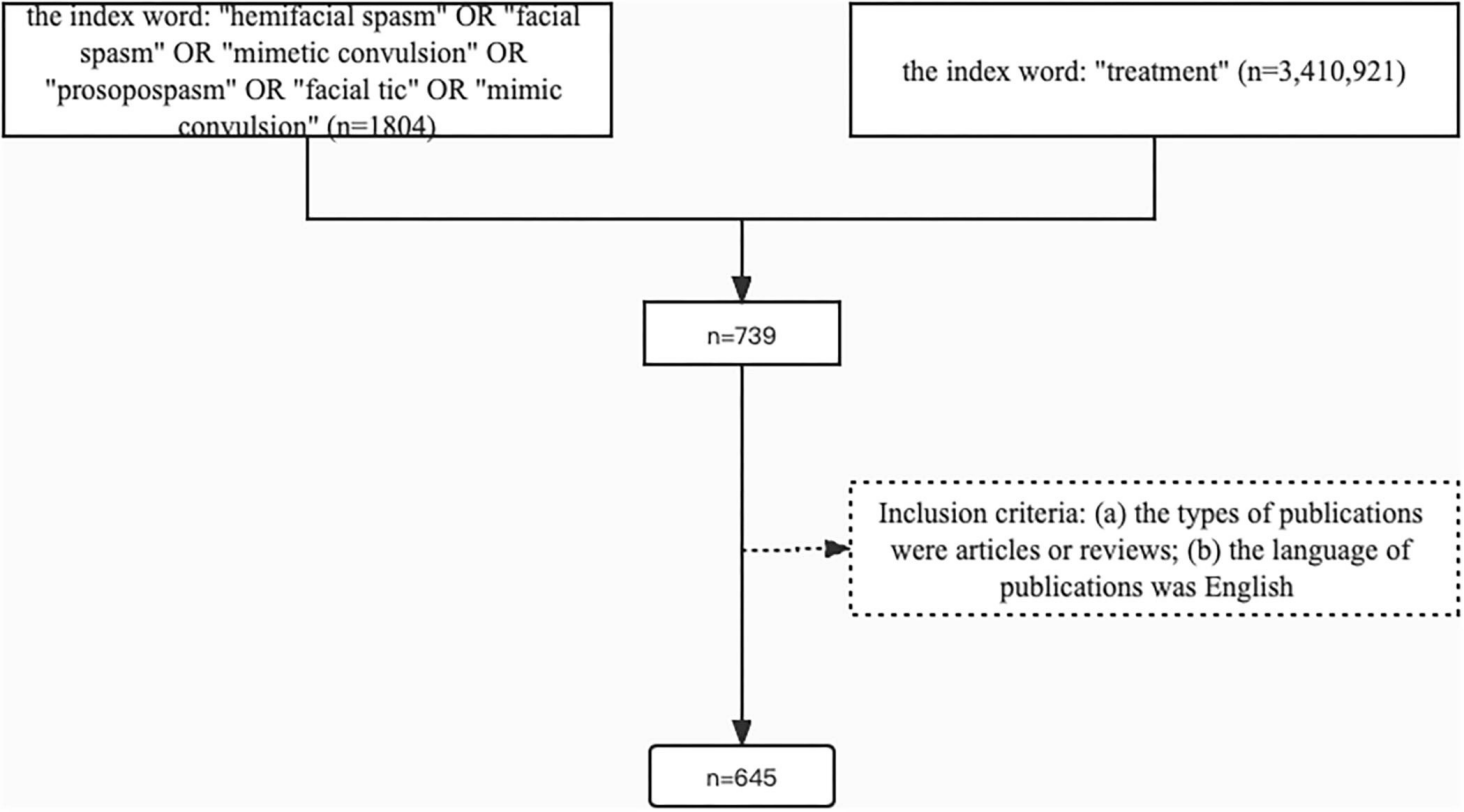
The preferred reporting items for systematic reviews and meta-analyses (PRISMA) protocol.

All data retrieval was based on the Preferred Reporting Items for Systematic reviews and Meta-Analyses (PRISMA) protocol (Figure 2), the documents were acquired from the Web of Science (WoS) database from inception to 23 April 2022. The retrieval strategy included 3 parts. Part 1: we searched the articles using the words “hemifacial spasm,” “facial spasm,” “mimetic convulsion,” “prosopospasm,” “facial tic,” and “mimic convulsion.” There were 1,804 records by this query. Part 2: we retrieved the articles using “treatment” as the index word, then 3,410,921 records were shown. Part 3: we combined two queries to focus on the treatment of HFS all this time. Ultimately, 739 articles were analyzed.

Inclusion criteria: (a) the types of publications were articles or reviews and (b) the language of publications was English. Finally, 645 articles were retrieved to conduct the bibliometric analysis.

### Analysis tool

Visualization of Similarities (VOS)viewer (version 1.6.18, Leiden University, Leiden, The Netherlands) was used to construct and visualize the bibliometric networks in this study. The program provides a viewer with a comprehensive and detailed examination of bibliometrics. For example, these networks may include journals, researchers, or individual publications, which may be constructed on the basis of citations, bibliographic coupling, co-citation, or co-authorship relationships (17). In the visualization using the VOSviewer, the attraction/repulsion values in the layout items were all set with attraction, 3/repulsion, −2 for clearer presentations.

## Results

### Analysis of annual publications

**Figure 3:**
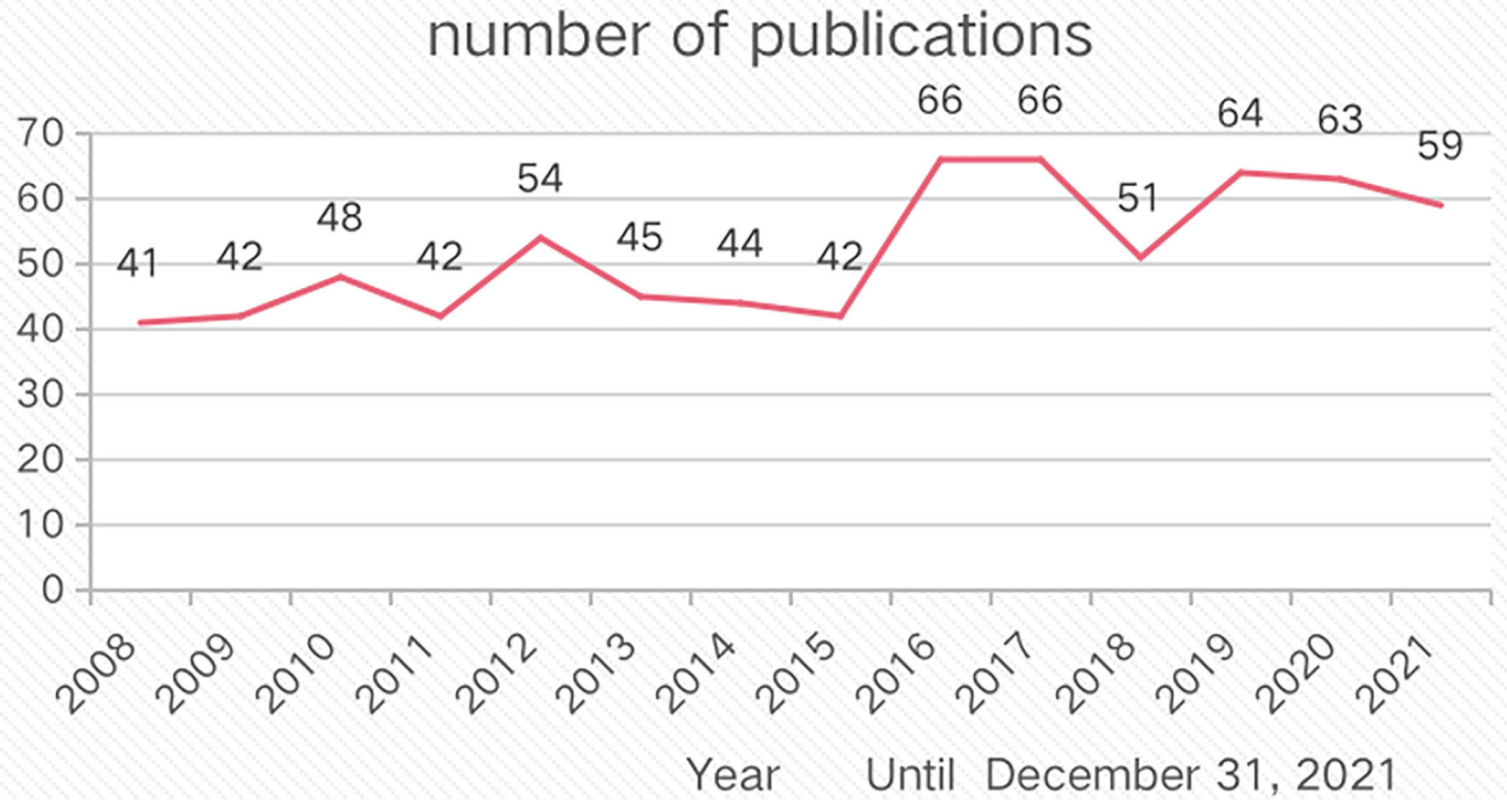
The annual publications from 2008 to 31 December 2021.

There were no relevant papers prior to 2008. Until 31 December 2021, the number of annual publications was fluctuating, but a gradual upward trend from only 41 in 2008 to 59 in 2021 (Figure 3).

### Analysis of authors

**Figure 4:**
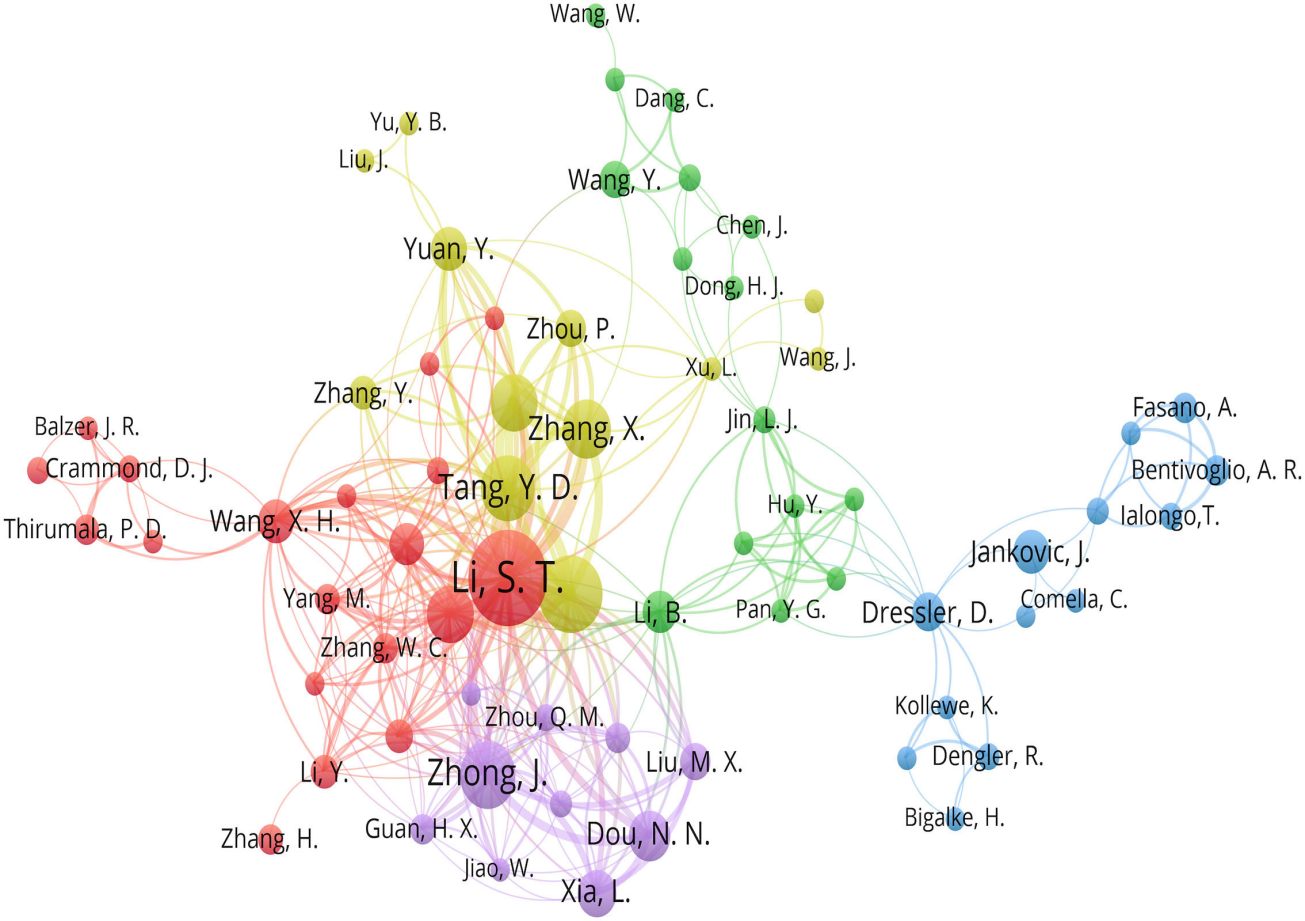
The links among the authors.

The 645 articles were written by 2,592 authors. In terms of the number of articles published, the top three most prolific authors were as follows: Li S. T. (45 publications), Zhu J. (29 publications), and Zhong J. (23 publications). The links among these articles are shown in Figure 4.

### Analysis of countries

**Figure 5:**
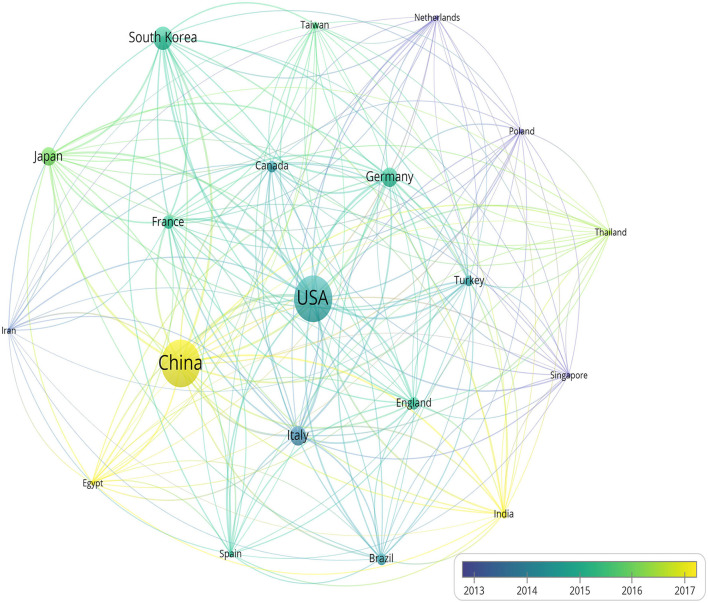
The map shows the average publication year of countries with yellow standing for later and purple standing for earlier.

With regard to the countries of the published literature related to the treatment of HFS, the top 10 most active countries are shown in Table 1. Furthermore, Figure 5 reveals the average publication year among these countries. The most prolific country was China, followed by the USA and South Korea.

**Table 1:** The top 10 countries that published the literature on the treatment of hemifacial spasm (HFS).

**Rank**	**Country**	**Papers**	**% (of 645)**
1	China	155	24.03
2	USA	154	23.88
3	South Korea	58	8.99
4	Germany	46	7.13
5	Italy	45	6.98
6	Japan	40	6.20
7	France	26	4.03
8	England	24	3.72
9	Canada	21	3.26
10	Brazil	21	3.26

### Analysis of keywords

Before analyzing the terms of the 645 articles, we created a thesaurus file so that some terms could be unified into 1 term. For example, “microvascular decompression,” “MVD,” and “decompression” were unified as “microvascular decompression surgery” and “stereotactic radiosurgery,” “gamma-knife radiosurgery,” and “surgical-treatment” were all unified as “surgery.”

**Figure 6:**
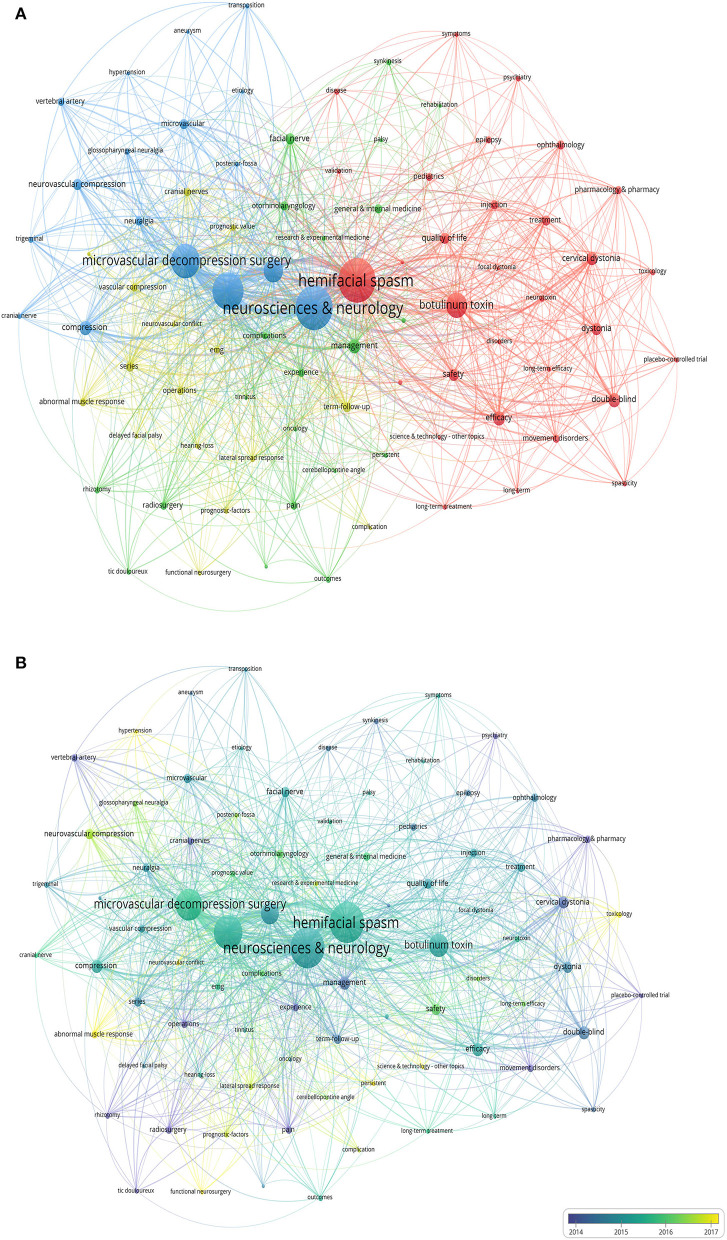
**(A)** The frequency of keywords in the published articles on the treatment of hemifacial spasm (HFS). **(B)** The map shows the average publication year of keywords with yellow standing for later and purple standing for earlier.

In total, 85 keywords out of the 2,499 terms were analyzed when we set “10” as the minimum occurrence of them. They were classified into 4 clusters with different colors according to the frequency (Figure 6A). Cluster 1 almost represented clinical studies; the frequency of hemifacial spasm was 420 times, followed by botulinum toxin (163 times). Cluster 2 represented clinical therapy; the most frequent term was management (69 times). Cluster 3 represented the etiology and operative treatment of HFS, the most frequent word was neurosciences and neurology (422 times), followed by surgery (320 times), microvascular decompression surgery (277 times), and trigeminal neuralgia (144 times). Cluster 4 represented pathogenesis consisting of abnormal muscle response, ephaptic transmission, lateral spread response, etc., series and term follow-up were the most frequent terms (38 times).

Figure 6B shows the keywords according to the average year of publications. Purple represents the keywords mentioned earlier while yellow represents later. Overall, treatment of HFS tends to be more scientific and cautious focusing more on pathogenesis and nature of the disease.

### Analysis of organizations

There were 832 organizations of 645 pieces of literature. We set “5” as the minimum documents of an organization, then 22 institutions were analyzed. The top 10 most productive organizations are shown in Table 2. Although the most productive institution was in China, eight out of 10 institutions were from other countries, such as South Korea, the USA, Germany, and Italy.

**Table 2:** The top 10 most prolific organizations.

**Rank**	**Organization (country)**	**Documents**	**%(of 645)**
1	Shanghai Jiaotong University (China)	56	8.68
2	Sungkyunkwan University (South Korea)	21	3.26
3	Baylor College Medicine (USA)	17	2.64
4	University of Pittsburgh (USA)	12	1.86
5	Hannover Medicine School (Germany)	12	1.86
6	Sichuan University (China)	11	1.71
7	Yonsei University (South Korea)	10	1.55
8	Mayo Clinic (USA)	9	1.40
9	Kyung Hee University (South Korea)	8	1.24
10	Universita Cattolica del Sacro Cuore (Italy)	8	1.24

### Analysis of journals

**Figure 7:**
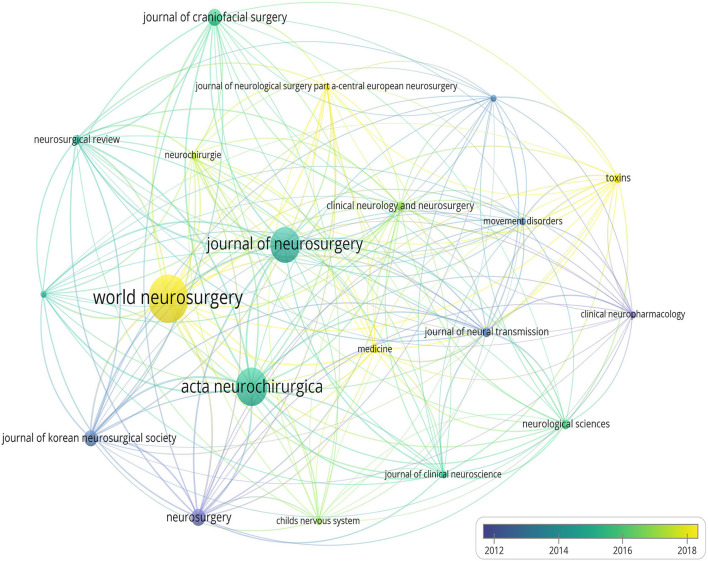
The map shows the average publication year of 20 journals with yellow standing for later and purple standing for earlier.

In total, 247 journals have delivered publications on the treatment of HFS. The 20 journals were finally extracted by setting “7” as the minimum number of documents of a source. Figure 7 shows the average publication year of 20 journals. Table 3 displays the top 10 most prolific journals on the treatment of HFS.

**Table 3:** The top 10 most prolific journals.

**Rank**	**Journal**	**Documents**	**IF (2020)**
1	World Neurosurgery	44	2.104
2	Acta Neurochirurgica	35	2.216
3	Journal of Neurosurgery	33	5.115
4	Neurosurgery	16	4.654
5	Journal of Craniofacial Surgery	16	1.046
6	Journal of Korean Neurosurgical Society	15	1.729
7	Toxins	9	4.546
8	Journal of Neural Transmission	9	3.575
9	Neurosurgical Review	9	3.042
10	Clinical Neurology and Neurosurgery	9	1.876

IF, impact factor.

## Discussion

The bibliometric analysis revealed that more researchers were concerned about the therapy of HFS. Recently, some investigators delivered articles about MVD in treating HFS (18–20), some studied the utilization of BoNT (21, 22), some researchers have published a randomized controlled trial on the treatment of HFS (23), and some employed a meta-analysis (24). However, a few papers were using VOSviewer to analyze the trends or hotspots of HFS. This study's purpose was to reveal an overview of treatment for HFS.

As for the annual publications, from only 41 papers in 2008 to 59 papers in 2021, the overall trend was on the rise. Since 2016, more than 60 articles were delivered mostly every year, in other words, 2016 was a turning point.

In terms of the authors included in this study, there were 8 clusters. Cluster 1 included Chen J., Dang C., Dong H. J., Hu Y., Jin L. J., et al. Cluster 2 included Albanese A., Bentivoglio A. R., Bigalke H., Comella C., Dressler D., Fasano A., et al., they were almost from Italy and Germany. Cluster 3 included Chen Z., Feng B. H., Hua X. M., Li S. T., Li X. Y., Ying T. T., Zheng X. S., Li Y., Zhang W. C., et al. Cluster 4 included Dou N. N., Guan H. X., Jiao W., Liu M. X., Sun H., et al. Cluster 5 included Balzer J. R., Habeych M., Sekula R. F., et al., most of them were from USA. Cluster 6 included Tang Y. D., Zhang X., Zhang Y., Zhou P., Zhu J., and Zhao H. Cluster 7 included Liu J., Yu Y. B., and Yuan Y. Cluster 8 included Liang W. B., Wang J., and Xu L. On the whole, Li S.T. appeared most frequently and always as the corresponding author. In addition, the Chinese group consisting of Li S. T., Ying T. T., Zheng X. S., Li Y., Zhang W. C., et al. was the most devoted to researching HFS (25, 26). Moreover, these active authors were from Shanghai and Beijing mostly, maybe cities' economics counted a lot. Then there was a lot of cooperation between Italy and Germany (27). China, the USA, and other countries could collaborate and probably put forward more original ideas.

In terms of the countries included in this study, China (24.03%) was the first that published the most papers about HFS's treatment, followed by the USA (23.88%), South Korea (8.99%), Germany (7.13%), and Italy (6.98%). Moreover, as shown in Figure 5, China, Egypt, and India announce more articles on the treatment of HFS in recent years.

According to the analysis of the keywords, we found that neurosciences and neurology, surgery, MVD, and BoNT were research focuses recently (Figure 6A). As shown in Figure 6B, neurovascular conflict, abnormal muscle response, functional neurosurgery, hypertension, toxicology, etc., were yellow. Nowadays, experts tend to have lucid knowledge of disease and implement scientific treatment.

As for organizations analyzed in this study, Shanghai Jiao Tong University published the most papers (8.68%), followed by Sungkyunkwan University (3.26%), Baylor College Medicine (2.64%), University of Pittsburgh (1.86%), Hannover Medicine School (1.86%), Sichuan University (1.71%), Yonsei University (1.55%), Mayo Clinic (1.40%), Kyung Hee University (1.24%), and Universita Cattolica del Sacro Cuore (1.24%). Generally speaking, most of them were universities, three were from the USA, three were from South Korea, and the most prolific institution was from China.

The 20 journals were extracted finally to research. From Figure 7, we noted that the journals about neurology were inclined to publish articles about HFS all along. Then in Table 3, *World Neurosurgery* (44 papers) published the largest number of articles, followed by *Acta Neurochirurgica* (35 papers), *Journal of Neurosurgery* (33 papers), *Neurosurgery* (16 papers), and *Journal of Craniofacial Surgery* (16 papers). However, the impact factor (IF) of these journals was under 6.

There are still some limitations to this study. First, the language of publications was restricted to English, so the results of the study cannot tell the whole story. Second, setting the minimum number of occurrences was so arbitrary that the outcomes would be affected. Third, the keywords left may not be representative enough due to the deviation from merging the synonyms.

Nevertheless, the research is significant and instructive in learning more directions of HFS, especially the treatment. In the future, I suggest that researchers could do a further study about it to announce a paper in journals with higher impact factors.

## Conclusion

In conclusion, the 645 articles were included in this study, then we found that the treatment of HFS caught more and more attention. Until 31 December 2021, the annual publications increased with a fluctuating tendency from 41 papers in 2008 to 59 papers in 2021. Moreover, the majority of authors who delivered the relevant papers (28–30) were from China. In addition, China was the most prolific country, and the USA followed.

From the analysis of keywords, neurosciences and neurology and surgery were the most mentioned. We investigated that the main treatment now was surgery. However, surgery always comes with complications, and the operation fee is relatively expensive, we hope that effective complementary and alternative therapies could be applied.

By searching the organizations, Shanghai Jiao Tong University was the first that published 56 papers, Zhong et al. have put forward the question that “is entire nerve root decompression necessary for HFS?” (31) and Zhu et al. discussed the surgical treatment of HFS (32), moreover, organizations from South Korea and the USA also contributed a lot.

About journals included, we found were almost surgical journals, but rarely journals of neurophysiology. *World Neurosurgery* preferred to publish articles about HFS. For example, surgical management (33), Gamma Knife Surgery (34), diagnosis and prognosis (35), and other aspects of HFS. *Acta Neurochirurgica* and *Journal of Neurosurgery* were followed. This reflects that MVD as the surgical method has been widely used as the most effective treatment for HFS since the 1980's (10). However, scholars have not focused on the neurophysiological aspects of the disease, and there are fewer relevant experimental studies. We should concentrate more on the functions and mechanisms of nerves and blood vessels associated with HFS and conduct more experiments to search for innovative treatments.

In the future, authors could conduct more high-quality studies through cooperation with other countries. Then the research of more therapies for the treatment of HFS can be done, such as complementary and alternative medicine.

## Data availability statement

The original contributions presented in the study are included in the article/supplementary material, further inquiries can be directed to the corresponding author.

## Author contributions

L-JF searched the literature, did the bibliometric analysis *via* VOSviewer, and finished this article. C-YW revised this manuscript. All authors approved the submitted version.

## Funding

This study was funded by the Natural Science Foundation of Zhejiang Province (No. LGF19H270003) and Young and Middle-Aged Clinical Famous TCM Doctors Project of Zhejiang Province [No. (2021)22].

## Conflict of interest

The authors declare that the research was conducted in the absence of any commercial or financial relationships that could be construed as a potential conflict of interest.

## Publisher's note

All claims expressed in this article are solely those of the authors and do not necessarily represent those of their affiliated organizations, or those of the publisher, the editors and the reviewers. Any product that may be evaluated in this article, or claim that may be made by its manufacturer, is not guaranteed or endorsed by the publisher.
